# Supplementary figures and images for: Life on the edge—a changing genetic landscape within an iconic American pika metapopulation over the last half century
Source: PeerJ. 2023 Sep 28;11:e15962. doi: 10.7717/peerj.15962 (PMC10542391; doi:10.7717/peerj.15962)

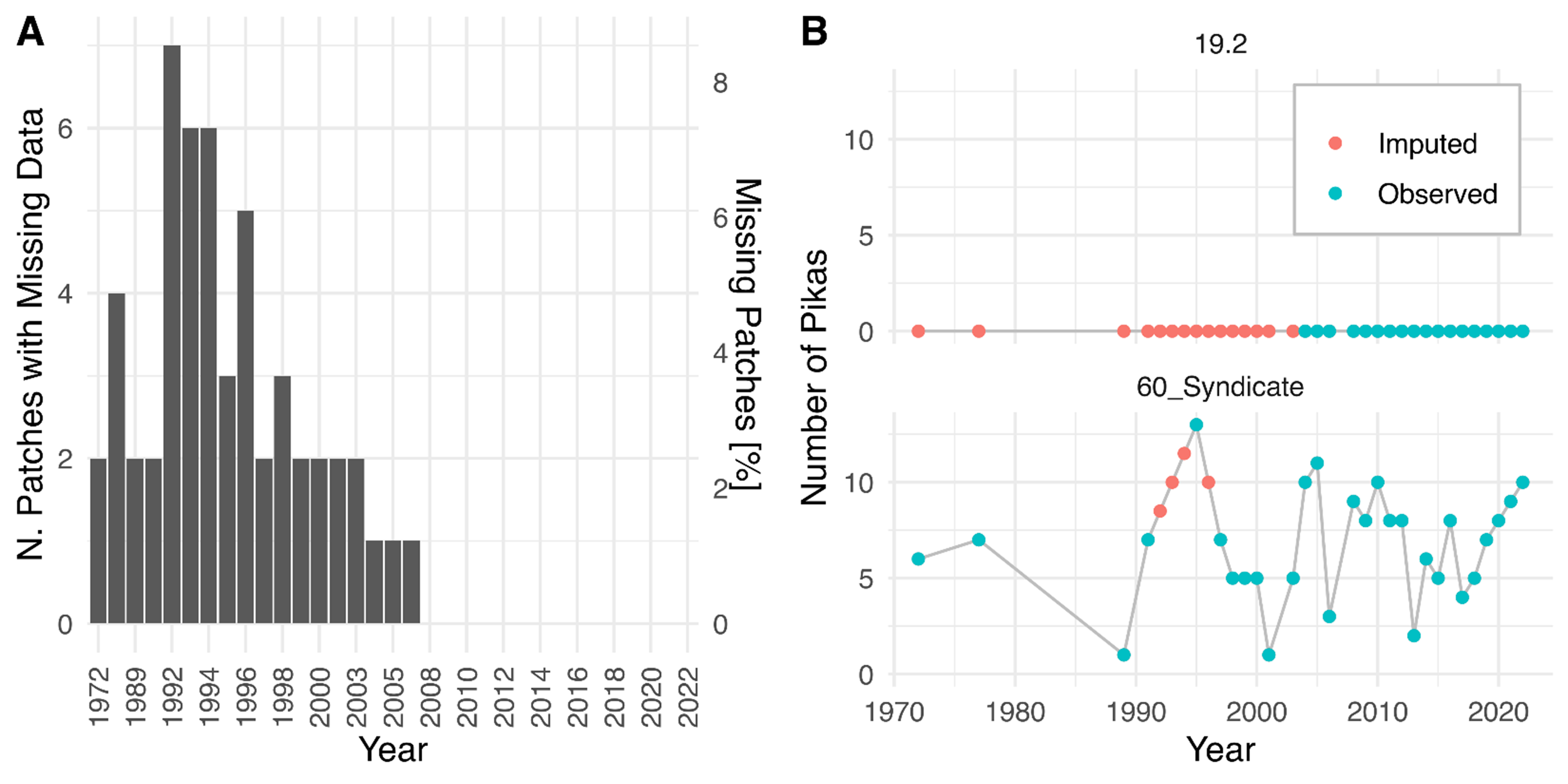

Supplement: Supplemental Information 1 — (A) Distribution of missing patch occupancy data over time. (B) Illustration of the linear imputation technique used to ameliorate the impact of missing data. See approxfun in [R], method = ‘linear’, rule = 2. [file peerj-11-15962-s001.png]

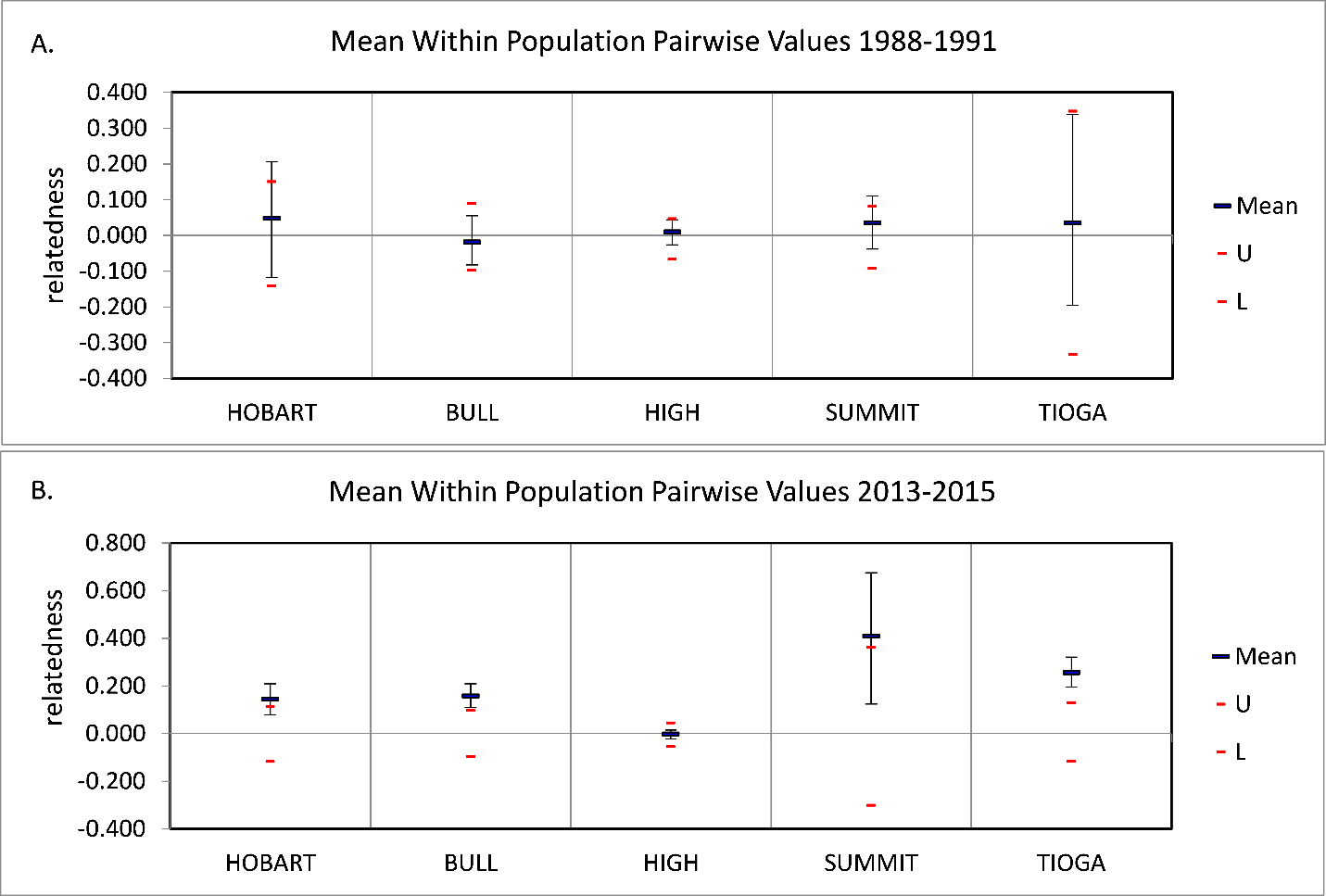

Supplement: Supplemental Information 2 — Upper (U) and lower (L) confidence limits bound the 95% confidence interval about the null hypothesis of ‘No Difference’ across the populations as determined by permutation (999 permutations, 1,000 bootstraps). [file peerj-11-15962-s002.png]

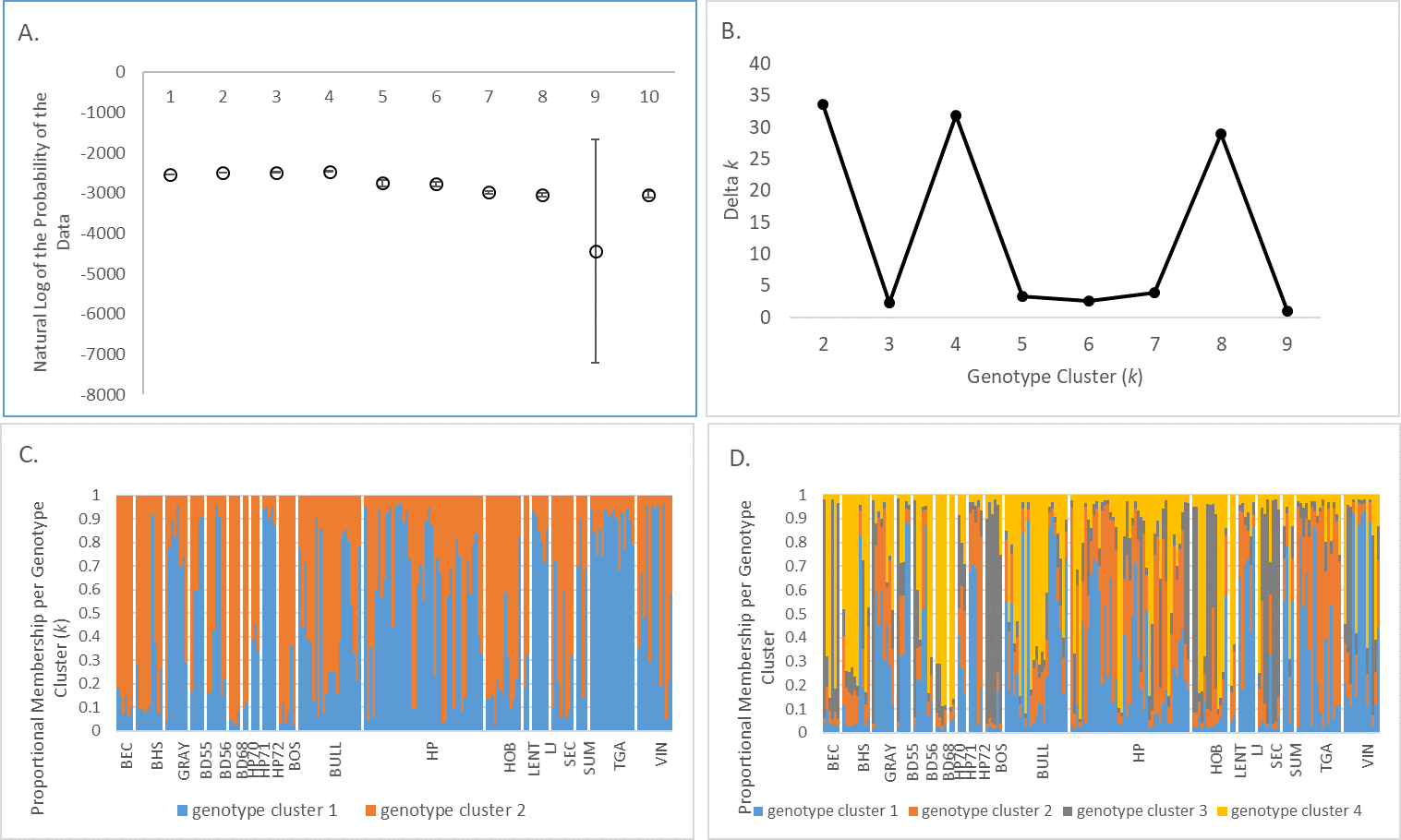

Supplement: Supplemental Information 3 — (A) The natural log of the probability of the data ((LnP(D)). (B) Delta k values showing support for k = 2 and k = 4. (C) Proportional membership per genotype cluster per individual for k = 2. (D) Proportional membership per genotype cluster per individual for k = 4. [file peerj-11-15962-s003.png]
