# Supplementary material for: Life on the edge—a changing genetic landscape within an iconic American pika metapopulation over the last half century
Source: PeerJ. 2023 Sep 28;11:e15962. doi: 10.7717/peerj.15962 (PMC10542391; doi:10.7717/peerj.15962)
Supplement: Supplemental Information 4 [file peerj-11-15962-s004.docx]

**Supplementary Tables**

Table S1. Collection date and location for pika specimens accessed from the Museum of Vertebrate Zoology (MVZ), University of California, Berkeley for BSHP 1948-1949 and BIH 1947.

| Cat_Num | Collectors | State_Prov | County | Specific_Locality | Date collected | Sex |
| --- | --- | --- | --- | --- | --- | --- |
| MVZ 107330 | Seth B. Benson (#10532) | Nevada | Mineral Co. | SE side Big Indian Mt. | 29-Jul-47 | female |
| MVZ 107331 | Seth B. Benson (#10533) | Nevada | Mineral Co. | SE side Big Indian Mt. | 29-Jul-47 | Male |
| MVZ 107332 | Seth B. Benson (#10534) | Nevada | Mineral Co. | SE side Big Indian Mt. | 29-Jul-47 | Female |
| MVZ 107333 | Seth B. Benson (#10536) | Nevada | Mineral Co. | SE side Big Indian Mt. | 29-Jul-47 | Male |
| MVZ 107334 | Seth B. Benson (#10537) | Nevada | Mineral Co. | SE side Big Indian Mt. | 29-Jul-47 | Female |
| MVZ 107335 | Oliver P. Pearson (#1054) | Nevada | Mineral Co. | SE side Big Indian Mt. | 29-Jul-47 | female |
| MVZ 107336 | Oliver P. Pearson (#1055) | Nevada | Mineral Co. | SE side Big Indian Mt. | 29-Jul-47 | female |
| MVZ 107337 | Oliver P. Pearson (#1056) | Nevada | Mineral Co. | SE side Big Indian Mt. | 29-Jul-47 | female |
| MVZ 107338 | Oliver P. Pearson (#1057) | Nevada | Mineral Co. | SE side Big Indian Mt. | 29-Jul-47 | female |
| MVZ 107339 | Oliver P. Pearson (#1058) | Nevada | Mineral Co. | SE side Big Indian Mt. | 29-Jul-47 | male |
| MVZ 135117 | Joye H. Severaid (#918) | California | Mono Co. | Bodie State Historical Park | 17-Jun-48 | female |
| MVZ 135118 | Joye H. Severaid (#919) | California | Mono Co. | Bodie State Historical Park | 17-Jun-48 | male |
| MVZ 135119 | Joye H. Severaid (#922) | California | Mono Co. | Bodie State Historical Park | 19-Jun-48 | male |
| MVZ 135120 | Joye H. Severaid (#923) | California | Mono Co. | Bodie State Historical Park | 19-Jun-48 | male |
| MVZ 135121 | Joye H. Severaid (#924) | California | Mono Co. | Bodie State Historical Park | 19-Jun-48 | female |
| MVZ 135122 | Joye H. Severaid (#925) | California | Mono Co. | Bodie State Historical Park | 19-Jun-48 | male |
| MVZ 135125 | Joye H. Severaid (#949) | California | Mono Co. | Bodie State Historical Park | 12-Jul-48 | female |
| MVZ 135126 | Joye H. Severaid (#950) | California | Mono Co. | Bodie State Historical Park | 12-Jul-48 | female |
| MVZ 135127 | Joye H. Severaid (#951) | California | Mono Co. | Bodie State Historical Park | 12-Jul-48 | female |
| MVZ 135133 | Joye H. Severaid (#992) | California | Mono Co. | Bodie State Historical Park | 15-Jul-48 | male |
| MVZ 135140 | Joye H. Severaid (#1013) | California | Mono Co. | Bodie State Historical Park | 8-Aug-48 | male |
| MVZ 135147 | Joye H. Severaid (#1078) | California | Mono Co. | Bodie State Historical Park | 23-May-49 | male |
| MVZ 135148 | Joye H. Severaid (#1084) | California | Mono Co. | Bodie State Historical Park | 29-May-49 | male |
| MVZ 135162 | Joye H. Severaid (#1111) | California | Mono Co. | Bodie State Historical Park | 24-Jun-49 | male |
| MVZ 135150 | Joye H. Severaid (#1078) | California | Mono Co. | Bodie State Historical Park | 23-May-49 | male |
| MVZ 135149 | Joye H. Severaid (#1088) | California | Mono Co. | Bodie State Historical Park | 10-May-49 | male |
| MVZ 135216 | Joye H. Severaid (#1241) | California | Mono Co. | Bodie State Historical Park | 29-Jun-49 | male |
| MVZ 135225 | Joye H. Severaid (#1316) | California | Mono Co. | Bodie State Historical Park | 8-May-49 | male |
| MVZ 135220 | Joye H. Severaid (#1305) | California | Mono Co. | Bodie State Historical Park | 18-Jun-49 | male |
| MVZ 135151 | Joye H. Severaid (#1089) | California | Mono Co. | Bodie State Historical Park | 1-Jun-49 | male |
| MVZ 135206 | Joye H. Severaid (#1203) | California | Mono Co. | Bodie State Historical Park | 4-Jun-49 | female |
| MVZ 135142 | Joye H. Severaid | California | Mono Co. | Bodie State Historical Park | X | male |

Table S2. Genetic metrics for all loci per location and time period; number of alleles (Na), allelic richness per locus, per location and time period (Rs), total allelic richness per locus across all time periods (Rt); expected heterozygosity (He), observed heterozygosity (Ho) and inbreeding coefficient (F_IS_). Significant F_IS_ are bolded and italicized.

| Locus |  | BIH 1947 | BSHP 1948-1949 | BSHP 1988-1991 | BSHP 2013-2015 | TOTAL  Na or Rt |
| --- | --- | --- | --- | --- | --- | --- |
| OCP4b | Na | 4 | 7 | 4 | 5 | 9 |
|  | Rs | 3.9 | 6.35 | 2.53 | 3.628 | 4.203 |
|  | He | 0.679 | 0.816 | 0.411 | 0.655 |  |
|  | Ho | 0.700 | 0.909 | 0.466 | 0.842 |  |
|  | F_IS_ | -0.033 | -0.117 | -0.135 | ***-0.286*** |  |
| OCP6b | Na | 4 | 6 | 3 | 4 | 8 |
|  | Rs | 4 | 4.828 | 2.988 | 3.036 | 3.376 |
|  | He | 0.647 | 0.774 | 0.645 | 0.641 |  |
|  | Ho | 0.333 | 0.714 | 0.680 | 0.734 |  |
|  | F_IS_ | 0.5 | 0.078 | -0.054 | -0.145 |  |
| OCP7 | Na | 4 | **-** | 5 | 4 |  |
|  | Rs | 3.345 | **-** | 5 | 4 |  |
|  | He | 0.712 | **-** | 0.608 | 0.523 |  |
|  | Ho | 0.667 | **-** | 0.716 | 0.57 |  |
|  | F_IS_ | 0.068 | **-** | -0.07 | 0.027 |  |
| OCP8 | Na | 5 | 6 | 6 | 7 | 12 |
|  | Rs | 4.9 | 5.196 | 5.126 | 4.572 | 5.59 |
|  | He | 0.789 | 0.779 | 0.757 | 0.701 |  |
|  | Ho | 0.700 | 0.619 | 0.743 | 0.755 |  |
|  | F_IS_ | 0.119 | 0.21 | 0.019 | -0.078 |  |
| OCP9b | Na | 4 | 5 | 4 | 4 | 6 |
|  | Rs | 3.8 | 4.397 | 3.12 | 3.036 | 3.371 |
|  | He | 0.537 | 0.768 | 0.675 | 0.617 |  |
|  | Ho | 0.500 | 0.864 | 0.595 | 0.764 |  |
|  | F_IS_ | 0.072 | -0.127 | 0.12 | -0.239 |  |
| OCP10 | Na | 3 | 6 | 4 | 5 | 8 |
|  | Rs | 2.995 | 5.205 | 3.515 | 2.579 | 3.964 |
|  | He | 0.568 | 0.774 | 0.613 | 0.246 |  |
|  | Ho | 0.400 | 0.250 | 0.307 | 0.214 |  |
|  | F_IS_ | 0.308 | ***0.683*** | ***0.501*** | 0.132 |  |
| OCP17 | Na | 1 | 7 | 6 | 8 | 12 |
|  | Rs | 1 | 5.51 | 4.603 | 3.856 | 5.204 |
|  | He | 0 | 0.735 | 0.699 | 0.499 |  |
|  | Ho | 0 | 0.474 | 0.76 | 0.444 |  |
|  | F_IS_ | NA | 0.362 | -0.087 | 0.109 |  |
| OCP21 | Na | 3 | 5 | 5 | 4 | 5 |
|  | Rs | 3 | 4.304 | 3.699 | 2.897 | 3.812 |
|  | He | 0.451 | 0.715 | 0.642 | 0.427 |  |
|  | Ho | 0.333 | 0.545 | 0.507 | 0.526 |  |
|  | F_IS_ | 0.273 | 0.241 | 0.212 | ***-0.232*** |  |

Table S3. Pairwise F_ST_ estimates among patch subpopulations for (A) 1988-1991, *p* = 0.0009, 1100 permutations, adjusted nominal level (5%) for multiple comparisons and (B) 2013-2015, p = 0.0002, 4200 permutations, adjusted nominal level (5%) for multiple comparisons, datasets. Significant values are bolded and italicized.

| A. | BEC | BD54-56 | BD68 | BULL | GRAY | HP | LJ | SCAN | SEC | SUM |
| --- | --- | --- | --- | --- | --- | --- | --- | --- | --- | --- |
| BECHTEL (BEC) |  |  |  |  |  |  |  |  |  |  |
| BODIE54-56 (BD54-56) | -0.080 |  |  |  |  |  |  |  |  |  |
| BODIE68 (BD68) | 0.012 | 0.116 |  |  |  |  |  |  |  |  |
| BULL RUN (BULL) | -0.098 | 0.001 | 0.019 |  |  |  |  |  |  |  |
| GRAY SHAFT (Gray) | 0.067 | 0.084 | 0.108 | 0.094 |  |  |  |  |  |  |
| HIGH PEAK (HP) | -0.038 | 0.019 | 0.019 | 0.011 | 0.036 |  |  |  |  |  |
| LUCKY JACK (LJ) | -0.047 | -0.044 | 0.041 | 0.023 | 0.075 | 0.035 |  |  |  |  |
| SCANDINAVIAN (SCAN) | -0.097 | 0.013 | 0.008 | 0.012 | -0.034 | -0.017 | -0.006 |  |  |  |
| SECURITY (SEC) | -0.012 | 0.069 | 0.096 | 0.060 | 0.153 | 0.039 | -0.055 | 0.039 |  |  |
| SUMMIT (SUM) | -0.078 | 0.016 | 0.035 | 0.025 | 0.015 | 0.007 | 0.034 | -0.021 | 0.018 |  |
| TIOGA SHAFT (TGA) | -0.104 | 0.026 | 0.131 | 0.047 | 0.164 | 0.098 | 0.000 | 0.078 | 0.078 | 0.0714 |

| B. | BEC | BHS | BD55 | BD56 | BD68 | BOS | BSH | BULL | HP70 | HP | HOB | LJ | SEC | SUM | TGA |
| --- | --- | --- | --- | --- | --- | --- | --- | --- | --- | --- | --- | --- | --- | --- | --- |
| BECHTEL (BEC) |  |  |  |  |  |  |  |  |  |  |  |  |  |  |  |
| BLACKHAWK (BHS) | 0.116 |  |  |  |  |  |  |  |  |  |  |  |  |  |  |
| BODIE55 (BD55) | 0.085 | 0.078 |  |  |  |  |  |  |  |  |  |  |  |  |  |
| BODIE56 (BD56) | 0.161 | 0.133 | -0.005 |  |  |  |  |  |  |  |  |  |  |  |  |
| BODIE68 (BD68) | 0.076 | 0.047 | 0.123 | 0.183 |  |  |  |  |  |  |  |  |  |  |  |
| BODIE OLD SHAFT (BOS) | 0.016 | 0.142 | 0.184 | 0.234 | 0.118 |  |  |  |  |  |  |  |  |  |  |
| BODIE SHAFT (BSH) | 0.164 | 0.063 | 0.084 | 0.158 | 0.133 | 0.204 |  |  |  |  |  |  |  |  |  |
| BULL RUN (BULL) | ***0.106*** | 0.052 | 0.073 | 0.115 | 0.108 | 0.157 | 0.050 |  |  |  |  |  |  |  |  |
| HIGHPEAK70 | 0.193 | 0.180 | 0.207 | 0.163 | 0.207 | 0.206 | 0.230 | 0.128 |  |  |  |  |  |  |  |
| HIGH PEAK (HP) | ***0.095*** | 0.050 | 0.083 | ***0.139*** | 0.100 | 0.124 | 0.001 | 0.045 | 0.054 |  |  |  |  |  |  |
| HOBART (HOB) | 0.083 | 0.059 | 0.097 | 0.158 | 0.110 | 0.118 | 0.108 | ***0.096*** | 0.138 | ***0.043*** |  |  |  |  |  |
| LUCKY JACK (LJ) | 0.187 | 0.107 | 0.033 | 0.130 | 0.190 | 0.255 | 0.020 | 0.085 | 0.156 | 0.083 | 0.155 |  |  |  |  |
| SECURITY (SEC) | 0.108 | 0.042 | 0.082 | 0.182 | 0.099 | 0.154 | 0.162 | 0.127 | 0.147 | ***0.116*** | 0.064 | 0.152 |  |  |  |
| SUMMIT (SUM) | 0.079 | 0.027 | -0.013 | 0.105 | 0.114 | 0.168 | 0.018 | 0.035 | 0.067 | 0.046 | 0.083 | -0.014 | 0.065 |  |  |
| TIOGA SHAFT (TGA) | ***0.164*** | ***0.094*** | 0.076 | 0.077 | 0.171 | 0.212 | 0.087 | ***0.069*** | 0.145 | ***0.085*** | ***0.122*** | 0.070 | 0.129 | 0.051 |  |
| VINDICATOR (VHO) | 0.129 | 0.080 | 0.076 | 0.155 | 0.148 | 0.175 | 0.042 | 0.023 | 0.081 | 0.031 | 0.089 | 0.093 | 0.123 | 0.069 | ***0.099*** |
